# Sensorimotor ECoG Signal Features for BCI Control: A Comparison Between People With Locked-In Syndrome and Able-Bodied Controls

**DOI:** 10.3389/fnins.2019.01058

**Published:** 2019-10-16

**Authors:** Zachary V. Freudenburg, Mariana P. Branco, Sacha Leinders, Benny H. van der Vijgh, Elmar G. M. Pels, Timothy Denison, Leonard H. van den Berg, Kai J. Miller, Erik J. Aarnoutse, Nick F. Ramsey, Mariska J. Vansteensel

**Affiliations:** ^1^UMC Utrecht Brain Center, Department of Neurology & Neurosurgery, University Medical Center Utrecht, Utrecht, Netherlands; ^2^Department of Engineering Science, University of Oxford, Oxford, United Kingdom

**Keywords:** brain-computer interface, implant, sensorimotor cortex, amyotrophic lateral sclerosis, brain stem stroke, electrocorticography, high-frequency band, low-frequency band

## Abstract

The sensorimotor cortex is a frequently targeted brain area for the development of Brain-Computer Interfaces (BCIs) for communication in people with severe paralysis and communication problems (locked-in syndrome; LIS). It is widely acknowledged that this area displays an increase in high-frequency band (HFB) power and a decrease in the power of the low frequency band (LFB) during movement of, for example, the hand. Upon termination of hand movement, activity in the LFB band typically shows a short increase (rebound). The ability to modulate the neural signal in the sensorimotor cortex by imagining or attempting to move is crucial for the implementation of sensorimotor BCI in people who are unable to execute movements. This may not always be self-evident, since the most common causes of LIS, amyotrophic lateral sclerosis (ALS) and brain stem stroke, are associated with significant damage to the brain, potentially affecting the generation of baseline neural activity in the sensorimotor cortex and the modulation thereof by imagined or attempted hand movement. In the Utrecht NeuroProsthesis (UNP) study, a participant with LIS caused by ALS and a participant with LIS due to brain stem stroke were implanted with a fully implantable BCI, including subdural electrocorticography (ECoG) electrodes over the sensorimotor area, with the purpose of achieving ECoG-BCI-based communication. We noted differences between these participants in the spectral power changes generated by attempted movement of the hand. To better understand the nature and origin of these differences, we compared the baseline spectral features and task-induced modulation of the neural signal of the LIS participants, with those of a group of able-bodied people with epilepsy who received a subchronic implant with ECoG electrodes for diagnostic purposes. Our data show that baseline LFB oscillatory components and changes generated in the LFB power of the sensorimotor cortex by (attempted) hand movement differ between participants, despite consistent HFB responses in this area. We conclude that the etiology of LIS may have significant effects on the LFB spectral components in the sensorimotor cortex, which is relevant for the development of communication-BCIs for this population.

## Introduction

The sensorimotor areas of the brain are a promising target area for the control of Brain-Computer Interfaces (BCIs) that aim to provide people with severe paralysis (locked-in syndrome, LIS) a channel for communication and environmental control. A wealth of electroencephalography (EEG) literature shows that movement is associated with decreases in the spectral power measured from the sensorimotor regions, specifically in the alpha/mu (8–12 Hz) and beta (13–30 Hz) frequency bands (Jasper and Penfield, 1949; Chatrian et al., 1959; Neuper and Pfurtscheller, 2001), commonly referred to as event-related desynchronization (ERD). The end of movement is typically associated with a short-lasting increase in beta power (event-related synchronization, ERS) (Pfurtscheller et al., 1996). These low-frequency band (LFB) EEG signal changes have been used to accomplish BCI control in able-bodied study participants (e.g., Wolpaw et al., 2000; Scherer et al., 2007; Blankertz et al., 2010; Thomas et al., 2013) as well as in several people with severe paralysis (e.g., Neuper et al., 2003; Kübler et al., 2005; Bai et al., 2010; Daly et al., 2013).

Over the past decades, subdural electrocorticography (ECoG) has received increasing attention as a signal acquisition technology for BCI purposes. In BCI research settings, ECoG signals are typically recorded from able-bodied people with refractory epilepsy who are temporarily fitted with these electrodes for clinical diagnostic purposes. Also in the ECoG-BCI research field, the sensorimotor cortex is recognized as an especially interesting source of signals to enable BCI control (Leuthardt et al., 2004; Hermes et al., 2011). Since ECoG allows one to capitalize on the detailed spatial organization of the sensorimotor cortex, multiple independent control signals may conceptually be extracted from this area using high-spatial-density ECoG grids (Slutzky et al., 2010; Branco et al., 2017). Importantly, research on implantable ECoG-based BCIs often focuses on movement-related increases in High-Frequency Band (HFB, >30 Hz) power (Crone et al., 1998; Chestek et al., 2013; Blakely et al., 2014; Branco et al., 2017), which are thought to reflect local processing and are considered to be more spatially focal than changes in LFB power (Miller et al., 2009; Hermes et al., 2012), potentially providing a more specific, and therefore more reliable, BCI control signal (Schalk and Leuthardt, 2011). Yet, it has also been shown that changes in LFB power may contribute to accurate decoding and reliable ECoG-BCI control (Schalk et al., 2007; Nakanishi et al., 2014; Vansteensel et al., 2016; Flint et al., 2017). Indeed, HFB activity in the sensorimotor cortex is thought to be highly correlated to lower frequency bands, with the amplitude of HFB activity being coupled to the phase of LFB oscillations during rest (Yanagisawa et al., 2012).

For sensorimotor BCIs to become of value for people with LIS, it is essential for them to be able to generate reliable responses in the sensorimotor cortex by imagining or attempting to move. Two common causes for LIS are amyotrophic lateral sclerosis (ALS) and brain stem stroke (Patterson and Grabois, 1986; Hayashi and Kato, 1989; Pels et al., 2017). Both of these conditions are associated with brain damage: ALS causes predominantly motor neuron loss in the motor cortex and spinal cord (see for review Agosta et al., 2018), whereas brain stem strokes that lead to LIS typically involve damage to the ventral pons (Patterson and Grabois, 1986; de Mendivil et al., 2013). It can be surmised that brain damage can result in changes in the sensorimotor neuroelectrical signal used for ECoG-BCI, potentially affecting the baseline spectral characteristics as well as the signal changes induced by attempts to move the hand. As stated above, most ECoG sensorimotor BCI research has so far been performed in able-bodied participants (i.e., people with epilepsy). Therefore, the extent to which the sensorimotor ECoG-BCI control signal is affected by ALS and by brain stem stroke remains unclear.

Within the Utrecht NeuroProsthesis (UNP) study, we aim to evaluate the usability of a fully implantable ECoG-based communication-BCI for daily life by people with LIS. Two participants with LIS (one due to ALS, see Vansteensel et al., 2016; one due to brain stem stroke) have been implanted with the system, including electrodes over the sensorimotor hand area. Interestingly, we noted differences between these participants in the spectral responses induced by attempted hand movement. Here, we aimed to evaluate whether or not the different ECoG response profiles observed in these two LIS participants are part of the normal distribution of response profiles in the general population. The implanted system provides extensive data on the controllability of the spectral signals, from which we can learn about the LFB and HFB spectral components in LIS. To investigate normalcy, ECoG data from the LIS participants need to be compared to those of a control group of able-bodied individuals. The only suitable population for this purpose consists of people who suffer from refractory epilepsy and temporarily receive subdural electrodes for diagnostic purposes. Therefore, we compared spectral oscillations during rest, and modulation of LFB and HFB features by (attempted) hand movement, between the LIS participants and able-bodied people with epilepsy.

## Materials and Methods

This study was carried out in accordance with the Declaration of World Medical Association (2013). Epilepsy participants gave written informed consent to participate in the study. LIS participants gave informed consent via a dedicated procedure (see Vansteensel et al., 2016 for details). The protocol was approved by the Medical Research and Ethics Committee Utrecht.

### Participants

#### LIS Participants

**FIGURE 1:**
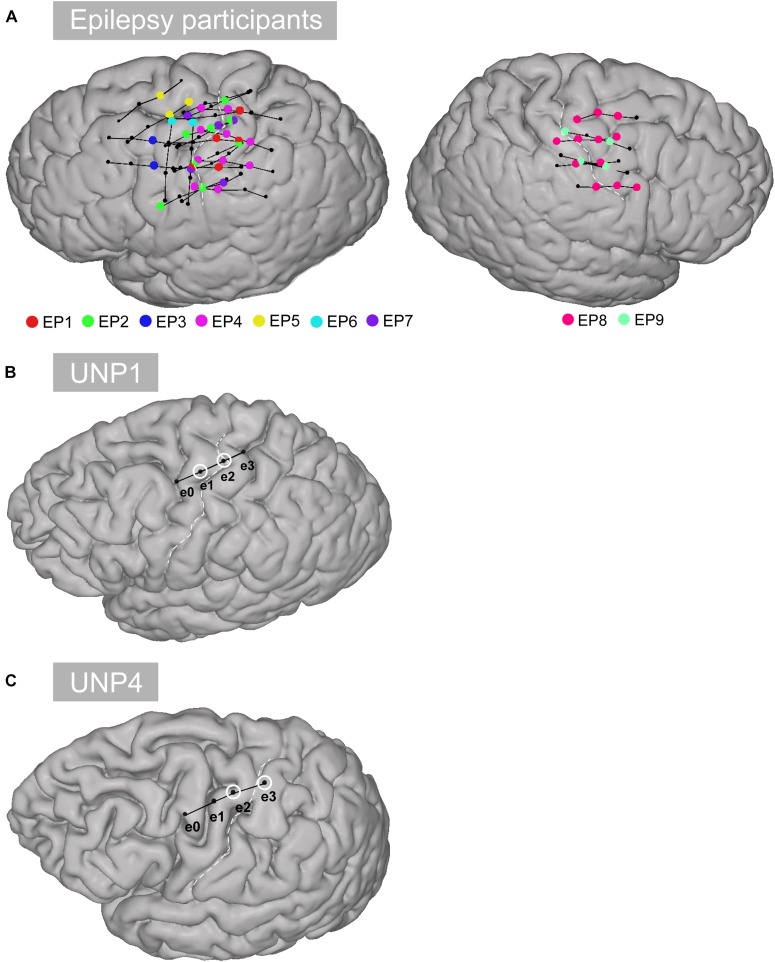
Location of the electrodes. **(A)** All selected rows of sensorimotor electrodes (black circles connected by black lines) in all epilepsy participants (EP1-EP9). Single electrodes showing a significant HFB response (*p* < 0.05) are indicated in color. **(B)** Sensorimotor electrode strip of UNP1. The electrodes marked with a white circle represent the most frequently studied electrode pair (e1-e2). **(C)** Sensorimotor electrode strip of UNP4. The electrodes marked with a white circle represent the most frequently studied electrode pair (e2-e3).

The first participant with LIS (UNP1; described in Vansteensel et al., 2016) is a woman, 58 years old at the time of informed consent in 2015, who had been diagnosed with ALS in 2008. She is severely paralyzed and anarthric as a result of her disease, but sensibility was intact during pre- and post-surgical neurological evaluation. For communication, she uses an eye gaze device for typing, as well as eye blinks, and more recently small movements of the corner of her mouth, to answer closed questions. In addition, she uses the implanted UNP device for communication. For that, she attempts to move the right hand and thereby generates reliable signal changes in the sensorimotor cortex, which are converted into “brain-clicks” to control communication software (Communicator 5, Tobii Dynavox, Danderyd, Sweden). The second participant with LIS (UNP4) is a woman who suffered from a brain stem stroke in 2004 and who has been severely paralyzed and anarthric as a result. Pre- and post-surgical neurological evaluation showed that sensibility was globally intact. She was 39 years old at the time of informed consent in August 2017. She uses a head switch to control scanning software for typing, and horizontal and vertical eye and head movements for answering closed questions. Both participants with LIS were implanted with subdural ECoG strips (Resume II, Medtronic, 4 circular contact points per strips, 1 cm inter-electrode distance, off-label use) over the left dorsolateral prefrontal cortex and over the hand region of the left sensorimotor cortex. The target location of the electrodes was determined using the results of an fMRI scan, which was conducted several weeks before surgery. Here, we describe results obtained from the sensorimotor electrodes (Figure 1). The participants have used the signal from sensorimotor electrodes for regular BCI training for 105 weeks (UNP1) and 61 weeks (UNP4), respectively, until the last datapoint included in this report. For analysis, the precise location of the subdural electrodes was assessed by co-registration of a post-operative CT scan with a pre-operative T1 MRI scan and a correction for brain shift, using the procedures described in Hermes et al. (2010) and Branco et al. (2018). Notably, two other LIS participants (UNP2 and UNP3) were included in this study, but died before surgery (Vansteensel et al., 2016).

#### Epilepsy Participants

Data from the participants with LIS were compared with data acquired from a group of 9 adult individuals (EP1-EP9; Table 1) with severe refractory epilepsy, who were temporarily implanted with subdural ECoG grids (Ad-Tech, Racine, United States, 1 cm inter-electrode distance) for clinical reasons. Individuals studied here had electrodes over the sensorimotor hand area (Figure 1), but the source of their epilepsy did not include this region in most cases. Note that in EP1 and EP8, the source of the epilepsy was located in the central areas, but the signal acquired during the Localizer task runs (see below) did not show interictal activity. All participants were MRI negative (i.e., no detectable structural or anatomical anomalies in the brain). Electrode localization was accomplished using a post-operative CT scan and a pre-operative T1 MRI scan, similarly as described for the LIS participants.

**TABLE 1:** Demographics of the able-bodied epilepsy participants.

**Participant#**	**Hemi**	**Focus**	**# 4-electrode rows**	**# HFB significant single electrodes**
EP1	L	L Central	3	5
EP2	L	L Superior Frontal	4	8
EP3	L	L Parietal-temporal-occipital	2	2
EP4	L	L Parietal	4	10
EP5	L	L Frontobasal	2	2
EP6	L	L Perisylvian	2	2
EP7	L	L Frontal Medial	4	5
EP8	R	R Central	4	12
EP9	R	R Frontal Parasagittal	2	4

*Hemi, hemisphere covered by ECoG electrodes; Focus, Location of the source of the epilepsy. Notably, on request of the journal, age and gender of the epilepsy participants are not included in this table.*

### Signal Acquisition

#### LIS Participants

The implanted electrodes (1 dorsolateral prefrontal strip and 1 sensorimotor strip) were connected via subdural leads to an implantable amplifier/transmitter device (Activa PC + S, Medtronic; Rouse et al., 2011; Afshar et al., 2013; off-label use), which was placed subcutaneously under the clavicle. This device is able to relay filtered spectral power signals from multiple bipolar pairs of electrodes to a tablet computer at a rate of 5 Hz for BCI control (see Vansteensel et al., 2016 for more details). In addition, the raw time-domain signal of a single bipolar electrode pair of each strip can be transmitted at 200 Hz. This setting is more energy consuming and is therefore only used during research visits. Here, we report on analyses of the time-domain signal recorded from single pairs of the sensorimotor electrode strip during repeated Baseline and Localizer tasks (see below) performed by the LIS participants.

#### Epilepsy Participants

Time-domain data from all implanted ECoG electrodes was continuously and simultaneously recorded using a clinical recording system (Micromed, Treviso, Italy, band pass filter 0.15–134.4 Hz) at a sampling frequency of 512 Hz. Epilepsy participants performed one or more runs of a Localizer task (see below), during which the ECoG signal was spliced to a computer running the BCI2000 software package (Schalk et al., 2004), where it was stored for offline signal processing.

### Tasks

#### Localizer Task

Locked-in syndrome participants periodically conducted a Localizer task that involved making repetitive attempted right hand movements or relaxing for alternating periods of 15 s. During each run of the Localizer task, the ECoG time-domain signal of one single electrode pair of the sensorimotor cortex strip was recorded (see Tables 2, 3 for the number of runs acquired per electrode pair). In both participants, one of the pairs was studied more frequently because it showed the most reliable responses (e1-e2 and e2-e3 for UNP1 and UNP4 respectively).

**TABLE 2:** For UNP1, the number of Localizer tasks runs acquired per bipolar electrode pair and percentage of these runs with a significant response in each of the three features studied.

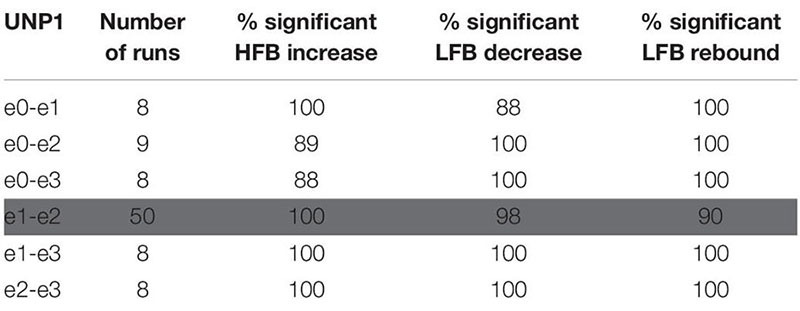

*The most frequently studied electrode pair is highlighted in gray.*

**TABLE 3:** For UNP4, the number of Localizer tasks runs acquired per bipolar electrode pair and percentage of these runs with a significant response in each of the three features studied.

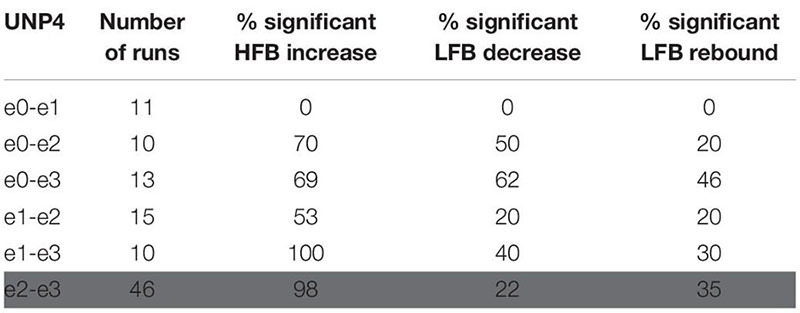

*The most frequently studied electrode pair is highlighted in gray.*

Five of the epilepsy participants performed a Localizer task that involved three different conditions in random order: rest, move (finger tapping of the hand contralateral to the implanted electrodes) and imagine (imagined finger tapping). Each trial had a duration of 15 s. For this study, only the move and rest conditions were analyzed. The other four epilepsy participants performed a task that contained alternating 30 s blocks of finger tapping and rest. Although the task performed by these four participants differed from the task performed by the other five participants in terms of the block-duration, the movement conditions of both tasks involved exactly the same action (finger tapping). Therefore, we did not distinguish between data acquired with the two paradigms. Instructions for the tasks were presented on a computer screen that was placed at the bedside (Presentation, Neurobehavioral Systems, Berkeley, CA, United States). Notably, the epilepsy participants were not involved in BCI feedback sessions before the Localizer task was acquired, except one participant, who did feedback sessions on 3 days.

#### Baseline Task

UNP1 and UNP4 regularly performed a Baseline task (2–5 min per run), in which they gazed at an image of a circle on a computer screen and were instructed to think of nothing in particular. Data from 52 to 32 runs (UNP1 and UNP4, respectively) recorded from the most frequently studied pair (e1-e2 for UNP1 and e2-e3 for UNP4) were analyzed in terms of spectral content of the LFB (see below).

### Signal Analysis: Presence and Consistency of LFB and HFB Features

#### LIS Participants

For every time sample of each time-domain data file, spectral power (6–100 Hz, 1 Hz bins) was computed using the real component of the convolution with a complex Gabor wavelet (Bruns, 2004; span 4 cycles at full width half max). The LFB and HFB responses over time where then computed as the sum over the log of the time varying amplitudes for two frequency ranges: 6–30 Hz (LFB) and 31–100 Hz (HFB). Subsequently, based on the well-described phenomena that occur in the sensorimotor cortex upon (attempted) movement (Jasper and Penfield, 1949; Chatrian et al., 1959; Pfurtscheller et al., 1996; Neuper and Pfurtscheller, 2001; Miller et al., 2007, 2010; Hermes et al., 2012; Vansteensel et al., 2016), we defined three movement-related signal features: (1) the increase in HFB (31–100 Hz; mean over time) power during active trials versus rest, (2) the decrease in LFB (6–30 Hz) power during active trials versus rest, and (3) the increase in LFB (6–30 Hz) power during the first 3 s following an active trial (i.e., the rebound period) versus rest. In all cases, rest was taken as the period after the rebound period (i.e., from 3 s after the onset until the end of a rest-trial). For each of these features, we computed the coefficient of determination (signed *R*^2^ value) per task run.

#### Epilepsy Participants

Data from all implanted subdual electrodes (excluding inter-hemispheric contacts and electrodes showing excessive noise or a flat signal, based on visual inspection) were common average re-referenced and evaluated in terms of response to the Localizer task, using the coefficient of determination (*R*^2^) between the mean 65–95 Hz power log amplitudes (maximum entropy method; Schalk et al., 2004) per-trial and the active and rest trials of the task design. To compare the data of the epilepsy participants with those of the LIS participants, we selected for each epilepsy participant one or more sets of four neighboring electrodes that were comparable to the electrode strips of the LIS participants, located over the superior part of the sensorimotor cortex and more or less perpendicular to the central sulcus where possible. The sets additionally contained at least one single electrode with a significant positive signed *R*^2^ (*p* < 0.05, Bonferroni corrected for multiple comparisons; see Table 1 for the number of “rows” selected for each epilepsy participant and the number of single HFB significant electrodes; see Figure 1 for the location of the selected rows). For each pair of selected rows of electrodes (i.e., six pairs per row), the LFB and HFB responses of the bipolar referenced signal were computed according to the same procedure as used for the participants with LIS.

#### Comparing LIS Participants With Epilepsy Participants

To investigate the co-occurrence of LFB with HFB power changes, we first determined the electrode pairs and runs that resulted in a significant change in the per-trial mean HFB power between active trials versus rest (significant positive signed *R*^2^ value, *p* < 0.05). This screening resulted in 18, 14, 5, 17, 1, 4, 27, 22, and 13 data points (electrode pairs and runs) for epilepsy participants EP1-9, and 89 and 79 data points for UNP1 and UNP4, respectively. For these pairs and runs, we computed, per participant, the mean signed *R*^2^ value for each of the three movement-related signal features, resulting in a single mean value per feature per participant. To evaluate whether the LFB responses of UNP1 and UNP4 fell in the normal range as observed in the epilepsy participants, we compared the mean signed R^2^ per LFB feature of UNP1 and UNP4 to the distributions of mean signed *R*^2^ values over epilepsy participants using *z*-scores.

### Signal Analysis: Oscillatory Components

To further investigate the spectral changes that lead to the LFB functional responses, we analyzed the spectral content of the Baseline task of UNP1 and UNP4, and of the active and rest periods of the Localizer task of all participants.

For each run of the Baseline task of UNP1 and UNP4 (only recorded from the most frequently studied electrode pair of each participant), we computed the spectral amplitude (1–100 Hz) over time. Then, the mean and standard deviation of the amplitude profile was computed per run.

For each active and rest period of the Localizer task of all participants (UNP1, UNP4, and epilepsy participants, only runs/electrode pairs with significant HFB response), we separated the oscillatory spectral peaks, which are attributed to rhythmic local field potential fluctuations, from the scale-free or fractal component by applying irregular-resampling auto-spectral analysis (IRASA; Wen and Liu, 2016). This procedure corrects for differences in mixed spectra profiles, for example caused by differences in electrode impedance over runs or in amplifiers between participants, and therefore allows for a direct comparison of the LFB oscillatory profiles. Rest and active data were split into smaller bins by applying a 3 s moving window with a step size of 1 s. Windows that overlapped the transition between active and rest or that included data from the rebound period (i.e., the first 3 s after the cue to stop moving) were excluded. The windows were irregularly resampled and for each resampling the auto-power spectra was computed, using methods described earlier (Wen and Liu, 2016). The fractal component was estimated by computing the median spectral profile for each of the resampled windows. The fractal component was subsequently subtracted from the non-resampled (or mixed) profile, resulting in the “oscillatory spectral profile” for each window. We then computed the means (over windows) of the mixed, fractal, and oscillatory profiles for both the active and rest task periods separately.

To compare the spectral content of the LFB of the LIS participants to those of the epilepsy participants, the difference between the normalized (z-scored over frequencies per participant) mean active and mean rest oscillatory profiles was computed and plotted. In this way the oscillatory functional changes of the UNP participants can be visually compared to those of the epilepsy participants. In addition, the UNP participants’ LFB (6–30 Hz) oscillatory profiles were compared per 1 Hz frequency bin with a one-sample student’s *t*-test to the mean epilepsy participant profiles.

## Results

### Presence and Consistency of LFB and HFB Features

**FIGURE 2:**
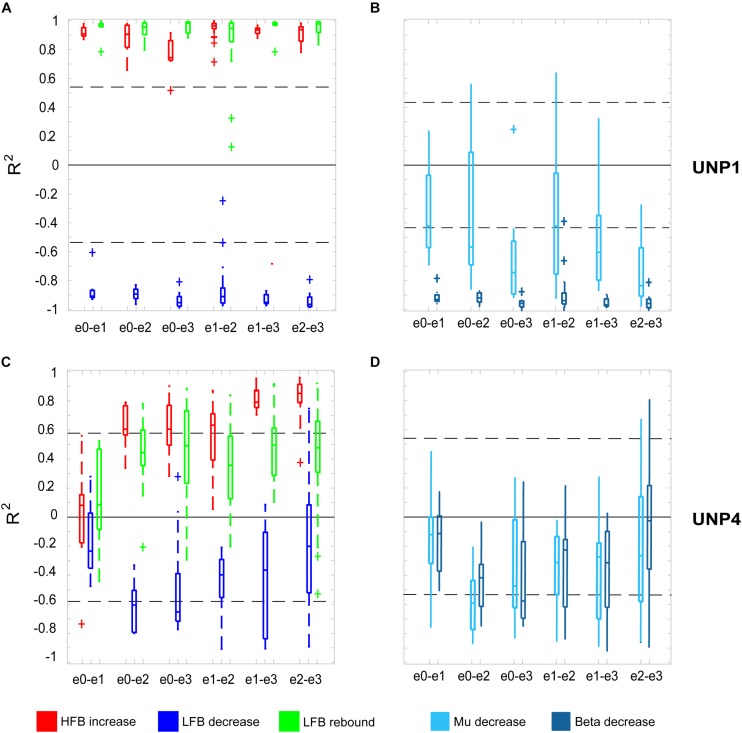
*R*^2^ values of HFB and LFB feature responses in UNP1 and UNP4. **(A,C)** Boxplot of the signed *R*^2^ values of each of the three studied features for each of the six electrode pairs of the sensorimotor electrode strip of UNP1 **(A)** and UNP4 **(C)**. Red, HFB increase; Blue, LFB decrease; Green, LFB rebound. The number of runs per pair is described in Tables 2, 3. The location of the electrodes is indicated in Figures 1B,C. **(B,D)** Boxplot of the signed *R*^2^ values of mu (8–12 Hz, light blue) and beta (13–30 Hz, dark blue) power decrease of UNP1 **(B)** and UNP4 **(D)**, per electrode pair. **(A–D)** The horizontal dashed lines indicate the lowest (absolute value) *R*^2^ value that was significant (*p* < 0.05) per participant. The solid black line indicates the value *R*^2^ = 0.

For all bipolar electrode pairs of UNP1 and for 5 out of 6 electrode pairs of UNP4, performance of the attempted hand movement Localizer task-induced a clear HFB response with high *R*^2^ values (median *R*^2^ value higher than 0.6; Figure 2A). For UNP1, these HFB responses were all accompanied by a consistent decrease in LFB power during the active trials and LFB rebound responses immediately thereafter. In contrast, the *R*^2^ values of the LFB decreases and LFB rebound responses of UNP4 were more variable and closer to zero (median typically smaller than 0.6; Figure 2C), even in the electrode pairs that showed the highest HFB *R*^2^ values. Notably, further splitting the LFB band into mu (8–12 Hz) and beta (13–30 Hz) frequency bands did not lead to larger or more consistent *R*^2^ values (Figures 2B,D).

**FIGURE 3:**
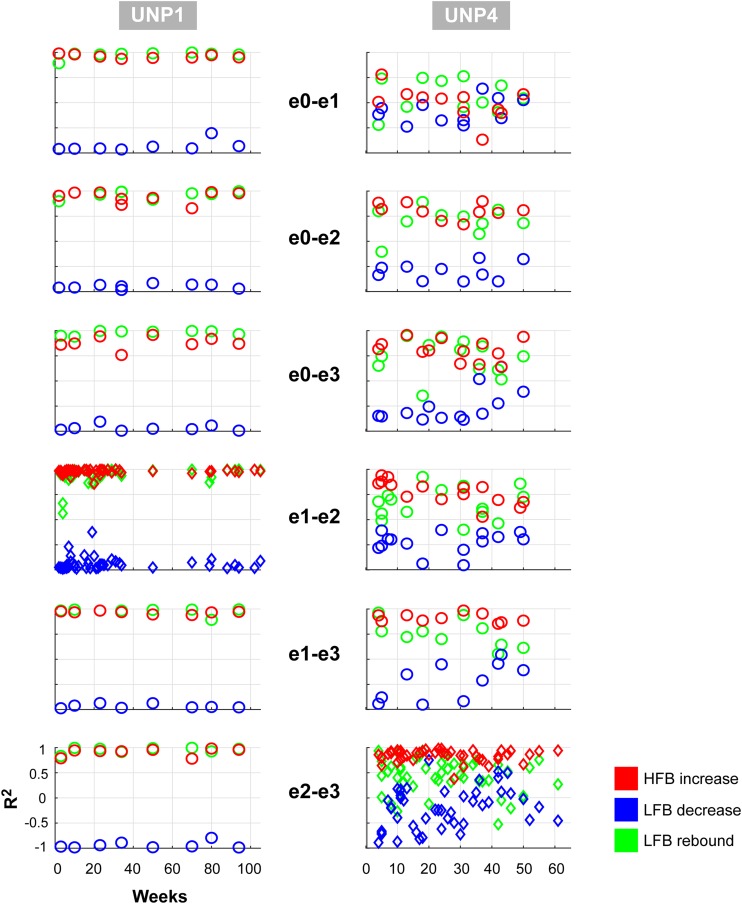
Consistency of LFB and HFB feature responses. Each panel shows, for each electrode pair of UNP1 **(left column)** and UNP4 **(right column)**, the signed *R*^2^ values of the three studied features for all runs acquired with that pair (1 symbol per run). Red, HFB increase; Blue, LFB decrease; Green, LFB rebound. Time on the *x*-axis indicates weeks since implantation. The panels with the diamond symbols indicate the data from the most frequently studied electrode pair.

We subsequently studied the consistency of the LFB and HFB response features. In UNP1, all pairs showed a significant HFB increase, LFB decrease and LFB rebound in 88% or more of the runs (Table 2). In contrast, for UNP4, for the two pairs that showed the most consistent HFB responses (e1-e3 and e2-e3), a significant LFB decrease or LFB rebound was obtained in 40% or less of the runs (Figure 3 and Table 3). Overall, a significant LFB response was obtained in only 62% or less of the runs.

**FIGURE 4:**
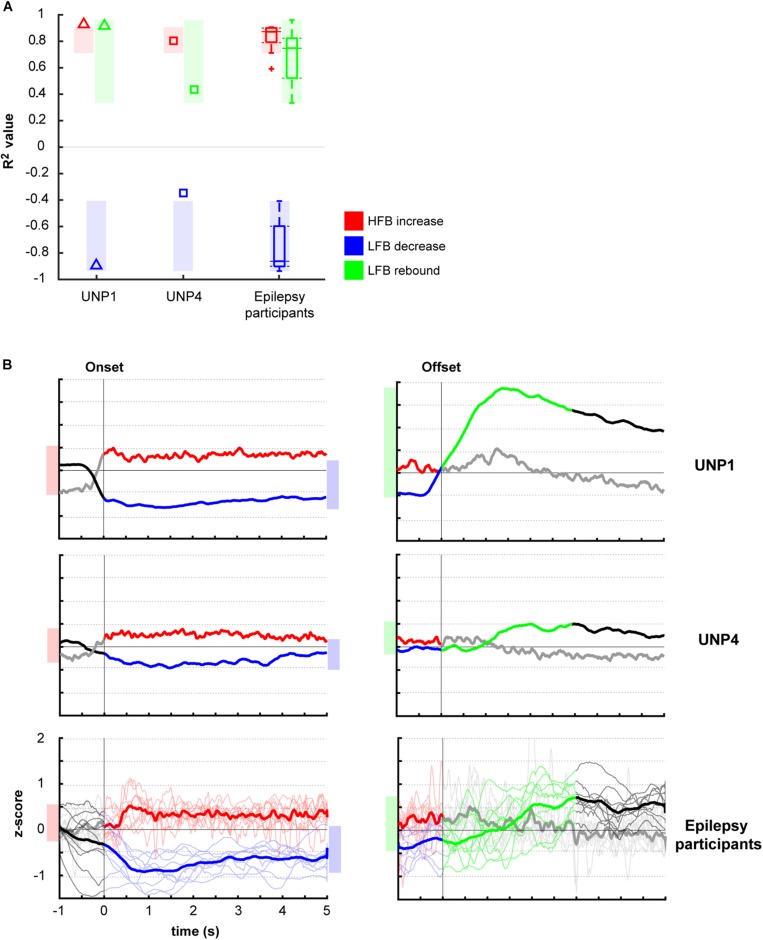
Comparison of *R*^2^ values of the HFB and LFB features between the LIS and epilepsy participants. **(A)** Per participant, the mean signed *R*^2^ value of each of the three features was computed for all runs/electrodes with a significant HFB response. For UNP1 (triangles) and UNP4 (squares), this mean signed *R*^2^ value is plotted. For the epilepsy participants, the distribution of the *R*^2^ values is given in a boxplot: horizontal line represents the median; the rectangle indicates the 50% of the distribution; the dashed lines indicate the maximum and minimum values; and the outliers are indicated by crosses “+.” The shaded areas indicate, per feature, the range of values observed among epilepsy participants. Red, HFB increase; Blue, LFB decrease; Green, LFB rebound. **(B)** Normalized (*z*-scored) power traces for each of the two UNP participants and the epilepsy participants (lower panels, where thin lines represent individual participants and the thick line the mean of all epilepsy participants). Left panels indicate HFB (red) and LFB decrease (blue) responses, locked to the onset of the active blocks in the Localizer task. Right panels indicate the LFB rebound responses (green), locked to the offset of the active blocks. Black and gray parts of the traces represent rest. Note that for the computation of the LFB rebound, only the first 3 s of the rest-trials were used, whereas for the HFB and LFB decrease responses, the entire active trial was included. Shaded bars next to the graphs are given for ease of comparison and indicate the variation of the (mean) responses (minimum until maximum value) for the given time window.

Comparison of the mean *R*^2^ values of the LFB decrease and LFB rebound responses of all runs/pairs with significant HFB changes between UNP1, UNP4, and the epilepsy participants revealed that the LFB decrease *R*^2^ value of UNP4 was smaller (i.e., closer to 0) than the range of LFB decrease *R*^2^ values observed in the epilepsy participants (*z*-score 2.14; Figure 4A). In addition, UNP1 and UNP4 had a rebound response that was among the 25% highest (UNP1; *z*-score 0.98) and 25% lowest (UNP4; *z*-score −1.04) values observed in able-bodied participants. Also looking at the (normalized) power traces reveals that UNP1 has large and UNP4 has small LFB responses (Figure 4B).

### Oscillatory Components

**FIGURE 5:**
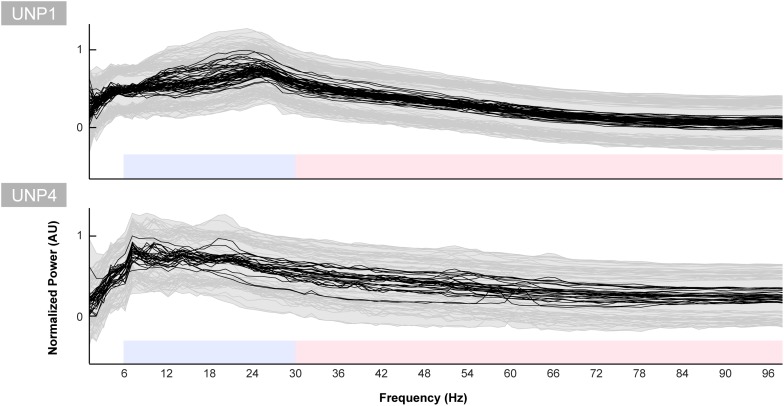
Baseline spectra for UNP1 and UNP4. Each black line indicates the normalized mean power spectrum over time of one Baseline task run of UNP1 **(upper panel)** and UNP4 **(lower panel)** as a function of frequency (Hz). Each subplot was normalized (between 0 and 1) separately to the maximum and minimum value of the black lines. The gray shading and lines indicates the standard deviation over time of each run. The blue and red blocks indicate the frequency ranges used for the LFB and HFB analysis, respectively.

**FIGURE 6:**
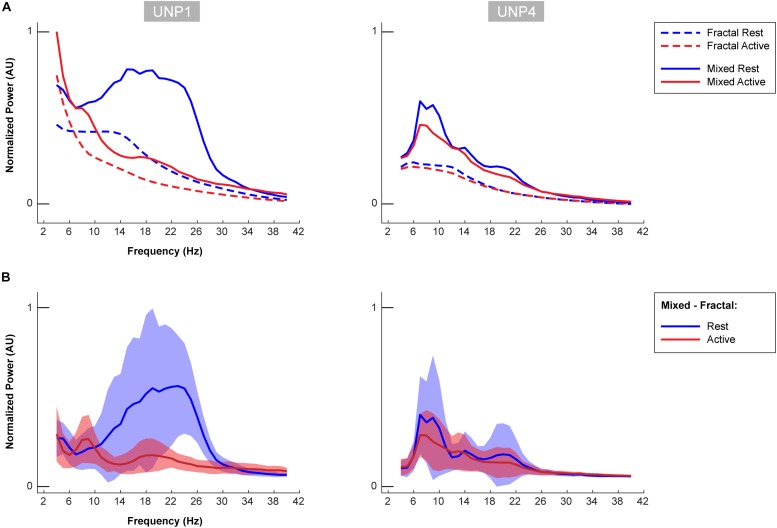
Oscillatory components of UNP1 and UNP4. **(A)** For UNP1 and UNP4, the mixed normalized power profile of the active and rest periods of the Localizer tasks are plotted in solid red and blue lines, respectively. The fractal normalized power profiles of the active and rest periods are given with dashed red and blue lines, respectively. The power profiles are plotted as a function of frequency (in Hz). Fractal and mixed components were normalized (between 0 and 1) by the maximum and minimum of the traces of both subjects (UNP1 and UNP4). **(B)** Subtraction of the fractal from the mixed spectra results in the oscillatory spectra. Blue solid line denotes the mean oscillatory spectral profile (across runs/electrode pairs) of the rest period. Red indicates the mean oscillatory spectral profile of the attempted hand movement (active) periods. The shaded regions indicate the standard deviation (across runs/electrode pairs) of each trace. Plots were normalized (between 0 and 1) by the minimum and maximum of the mean ± standard deviation traces of both subjects (UNP1 and UNP4).

The mean spectra of the Baseline runs of electrode pair e1-e2 of UNP1 showed a broad peak between ∼10 and 28 Hz (Figure 5). In addition, also the mixed spectra of the rest periods of the Localizer task (all runs/pairs with a significant HFB response) showed this clear and broad peak (Figure 6). Subtraction of the fractal component revealed the presence of a clear oscillatory component between 10 and 28 Hz that disappeared during the active periods of the Localizer task. In frequencies lower than 10 Hz, however, attempted hand movement did not seem to generate a decrease in power.

For UNP4, the Baseline spectra (electrode pair e2-e3) and the mixed spectra of the rest periods of the Localizer task (all pairs/run with a significant HFB response) showed a peak between 6 and 22 Hz (Figure 6). The oscillatory spectral profile, however, clearly peaked between 6 and 10 Hz during rest and, on average, did not show distinct peaks in low frequencies above 10 Hz. Importantly, the 6–10 Hz peak was hardly affected by attempted movement.

**FIGURE 7:**
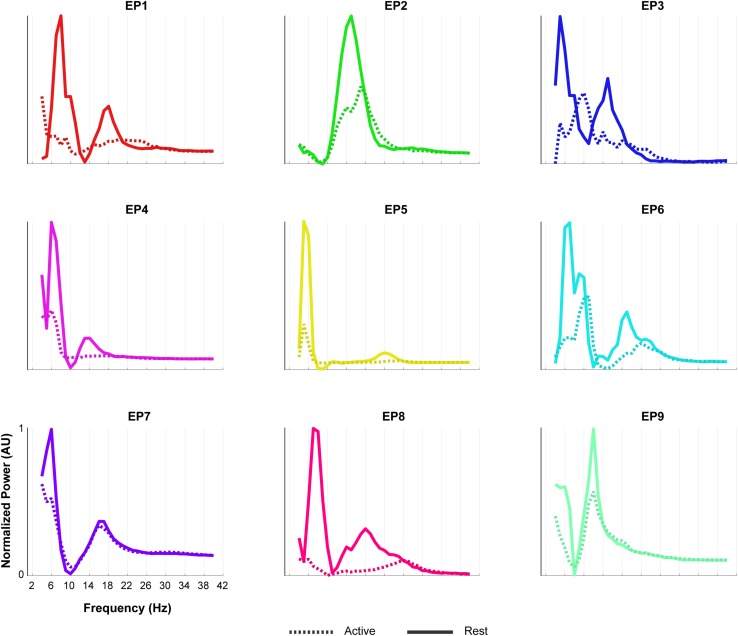
Oscillatory components of epilepsy participants. For each of the epilepsy participants (EP1-9), normalized power of the oscillatory component of the rest (solid line) and the active (dashed line) period of the Localizer task is given. In order to compare the shape of the traces across subjects, each plot was normalized (between 0 and 1) separately by the minimum and maximum of each subjects trace. Note the presence of two LFB peaks during rest in most participants and the decrease in power during the active period.

**FIGURE 8:**
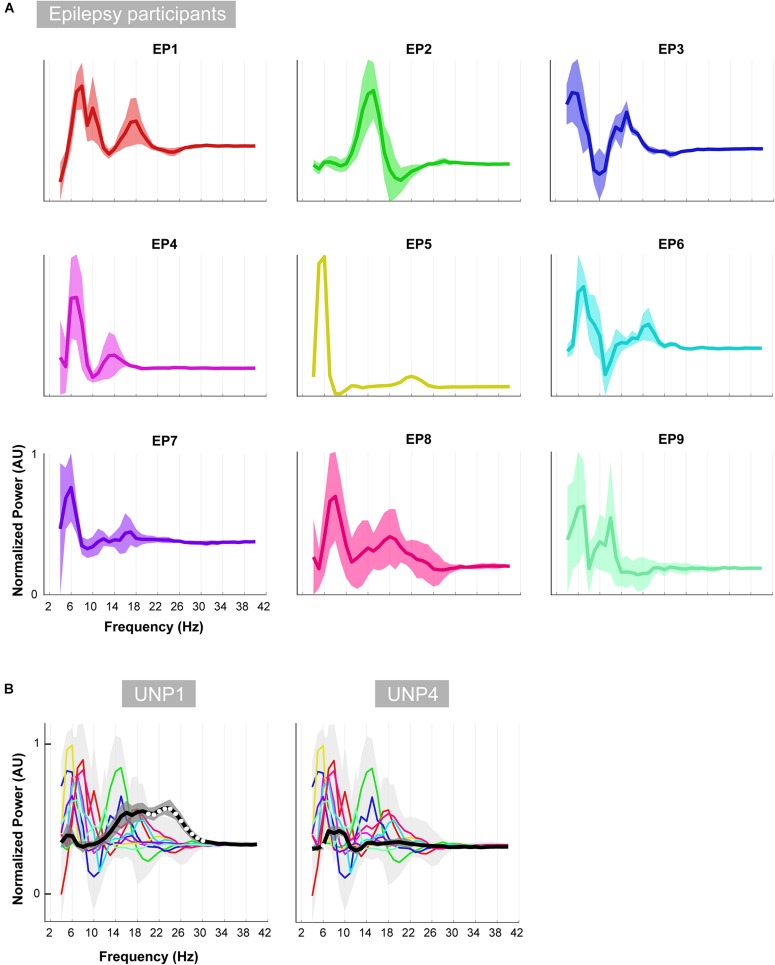
Oscillatory differences. **(A)** For each of the epilepsy participants (EP1-9), the mean difference between the active and rest oscillatory profile across runs/electrode pairs is given. Shaded regions indicate the standard deviation. In order to compare the shape of the traces across subjects, each plot was normalized (between 0 and 1) separately by the minimum and maximum of each subjects trace. **(B)** Each colored line indicates the difference between the active and rest oscillatory profile of each epilepsy participant. Light gray shading indicates standard deviation. For comparison, the mean difference profile (across runs/electrode pairs) of UNP1 (left) and UNP4 (right) is plotted in black, with dark gray shading indicating standard deviation. All traces were normalized (between 0 and 1) by the minimum and maximum of the mean ± standard deviation of the traces of all subjects (EP1-EP9, UNP1 and UNP4). Frequency bins that showed significant (one-sample *t*-test; *p* < 0.05; Bonferroni corrected) difference between UNP participants (black line) and epilepsy participants (colored lines) are indicated with white dots.

The oscillatory spectral profiles of the epilepsy participants typically included two peaks during the rest periods of the Localizer task, one between ∼5 and 8 Hz, and one between ∼12 and 22 Hz (Figure 7). During movement, power in both peaks decreased strongly (compare Figure 7 with Figure 8A).

Visual comparison, between UNP1 and the epilepsy participants, of the difference between the active and rest spectra showed the absence (in UNP1) of task-modulation in the <10 Hz range and large amplitude differences between rest and attempted movement in a beta range that was broader than observed in the epilepsy participants. Indeed, a one-sample *t*-test showed a significantly higher normalized mean amplitude in UNP1 for the range 19–30 Hz (*p* < 0.05, Bonferroni corrected; Figure 8B). For UNP4, visual comparison revealed some level of task-related modulation in the 6–10 Hz range. The beta range seemed entirely devoid of task-related changes in power, in contrast to what was observed in the epilepsy participants (except EP5, who also showed only minor modulation in the beta range).

## Discussion

We investigated the characteristics of three different sensorimotor ECoG signal features that are regularly targeted for BCI control purposes, and compared these features between two participants with LIS from different etiologies and able-bodied participants with epilepsy. Our data reveal important differences between participants in the LFB changes generated in the sensorimotor cortex by (attempted) hand movement, despite consistent HFB responses in this area.

Two participants with LIS, UNP1, and UNP4, received a fully implantable ECoG-based BCI system, including a subdural electrode strip over the sensorimotor hand area, as part of a study that aims to evaluate the usability of the BCI for day-to-day communication at home. In both participants, most electrode pairs of the sensorimotor electrode strip showed a clear HFB power increase upon attempted hand movement. Yet, only for UNP1 this HFB response was consistently accompanied by a movement-related LFB decrease and a post-movement LFB rebound response. In UNP4, changes in LFB power were smaller on average and electrode pairs and runs that responded with a consistent HFB increase in UNP4 did not necessarily display a significant LFB decrease or rebound. These data suggest that there are important differences in the sensorimotor ECoG signal features between the individuals with LIS. Comparison of the LFB responses of UNP1 and UNP4 with those of a group of epilepsy participants revealed that electrode pairs and runs that display a significant increase in HFB power during (attempted) movement show, on average, a strong LFB decrease and LFB rebound in the epilepsy participants and in UNP1. In UNP4, however, the mean LFB decrease *R*^2^ value was smaller (i.e., closer to 0) than the range of LFB decrease *R*^2^ values observed in the epilepsy participants.

To further investigate the LFB responses, we examined the spectral changes underlying the LFB functional responses by computing the oscillatory spectral profiles during (attempted) hand movement and during rest, for all electrode pairs and runs with a significant HFB response. In the epilepsy participants, two peaks were typically observed that were both strongly modulated by hand movement: one around 5–8 Hz and one around 12–22 Hz. Importantly, the center frequency of the 5–8 Hz peak was lower than classically reported in scalp EEG studies for the alpha/mu band oscillations over the central areas (Chatrian et al., 1959). This finding corresponds with results of a comprehensive investigation of the dominant frequencies in baseline ECoG recordings (Groppe et al., 2013). In that study, it was demonstrated that while beta (centered around 17 Hz) is clearly present in the ECoG measured from sensorimotor areas, alpha/mu activity is hardly observed in this region, and theta activity (4–8 Hz) is a dominant feature throughout the brain. Since hand movement by able-bodied people induced a clear power attenuation in the 5–8 Hz range in our data, it may be hypothesized that this ECoG feature represents the same phenomenon as described by the alpha/mu band in EEG studies. In contrast to the oscillatory spectral profiles of epilepsy participants, the rest profile of UNP1 showed a single, broad peak spanning a large part of the LFB range. In the beta band, power was strongly modulated by attempted movement, but not in lower frequencies. In UNP4, LFB oscillatory activity was limited to a peak in the 6–10 Hz range, which, however, was only minimally modulated by attempts to move the hand.

Our data suggest that the neuroelectrical ECoG features in the sensorimotor cortex of people with LIS display important differences with those of able-bodied people with epilepsy. Whereas both people with LIS were able to generate the well-described changes in the HFB upon attempted movement, LFB features were atypical. It could be surmised that the differences between the LIS participants and the epilepsy participants were related to the large differences in BCI training: both LIS participants have been involved in BCI feedback sessions for many months, whereas ECoG recordings in epilepsy patients typically last about one week and most participants did not have feedback training before the Localizer task was acquired. Importantly, however, already in the first measurements of the LIS participants, the differences in the LFB features were clear (Figure 3). In addition, we recently showed that long-term BCI use is not associated with significant changes in the control signal (Pels et al., 2019). Therefore, we believe that differences in training are unlikely to be associated with the atypical LFB features we observed. Instead, we postulate that these findings are suggestive of an effect of the underlying etiology of LIS on LFB baseline power in the sensorimotor cortex and on the modulation thereof by attempted hand movement. One important difference between ALS and brain stem stroke is the temporal aspect of the condition that leads to the locked-in state: whereas brain stem stroke is an event that suddenly disrupts motor function, ALS is a progressive disease that causes increasing muscle function loss over the course of months or years. Conceptually, there may be more room and time for adaptive changes and compensation in the case of ALS than for brain stem stroke, but it could also be reasoned that the longer period UNP4 has been in the locked-in state would allow for these changes. Alternatively, the difference in the location of the damage to the brain may underlie the different brain signal features we observed. Below, we discuss our results in the context of reported effects of brain stem stroke and ALS on the neuroelectrical signal.

### Amyotrophic Lateral Sclerosis

Previous reports about the effects of ALS on the neuroelectrical signal are relatively scarce and equivocal. Some EEG studies have reported a decrease in baseline alpha (Mai et al., 1998; Santhosh et al., 2005) and theta (Jayaram et al., 2015) power in people with ALS compared to controls, while others indicated heightened baseline alpha/mu power (Iyer et al., 2015; Maležič et al., 2016) or no difference (Geronimo et al., 2016). In contrast to some EEG studies where modulation of the alpha/mu frequency band has been used for sensorimotor BCI control by individuals with ALS (Wolpaw et al., 1997; Kübler et al., 2005), we observed that frequencies lower than 10 Hz were not modulated by attempted hand movement in UNP1. Possibly, the level of disease progression is related to this difference: the participants in the earlier studies were more recently diagnosed with ALS and still had some control over their limbs. Other possible explanations for the absence of modulation in frequencies lower than 10 Hz in UNP1, in the presence of clear ERD in higher LFB frequencies, is that the specific cortical area from which the electrode strip of UNP1 measures does not show this modulation or that there are individual differences in the modulation of this frequency range. Indeed, mu and beta desynchronization in the EEG signal often co-occur, but beta ERD may be observed without accompanying mu desynchronization and the two bands are thought to have distinct functional significance (Pfurtscheller, 1981).

Recently, Proudfoot et al. (2017) reported stronger movement-induced beta ERD in MEG recordings of people with ALS, compared to healthy controls. This result did not agree with earlier studies showing similar-sized beta ERD between patients and healthy controls (Riva et al., 2012) or a decreased ERD in patients (Kasahara et al., 2012), but does seem to correspond with our data of UNP1. Whereas the *R*^2^ value of the total LFB decrease (6–30 Hz) of HFB significant channels/runs of UNP1 was within the range observed for the epilepsy participants, the results of the oscillatory component analysis suggest that the LFB response of UNP1 is largely driven by frequencies larger than 10 Hz, rather than by a combination of the 5–8 and 12–22 Hz changes, as was typically observed in the epilepsy participants.

With respect to the beta rebound, there is evidence for a delayed (Proudfoot et al., 2017) or a smaller amplitude response (Riva et al., 2012) in people with ALS, but also for a preservation of beta ERS (Bai et al., 2010). In our study, the *R*^2^ value of the LFB rebound of UNP1 was among the 25% highest values observed in the epilepsy participants, which agrees with a preservation of beta ERS. Since we used the aggregate signal of the entire 3 s window post-movement-termination, we cannot draw any conclusions on the presence or absence of a delay in the LFB rebound response.

Previously, Jayaram et al. (2015) reported elevated baseline EEG HFB power in people with ALS, except in one, most severely motor impaired, patient. In another study, baseline ECoG HFB power was studied in an individual with ALS who transitioned from LIS to Complete LIS (CLIS). HFB power was present during LIS, but the transition to CLIS was accompanied by a sharp drop in this feature (Bensch et al., 2014). Although we did not quantify baseline HFB power in this study, the earlier findings on measurable HFB signal in people with ALS are in general agreement with our findings of clear and consistent HFB responses to attempted movement in UNP1. Whether or not a decrease in baseline HFB power is a general aspect of very late stages of ALS and the transition to CLIS (or of possible changes in alertness or cognition in this state) remains to be determined.

Taken together, our results indicate that HFB and LFB responses may be preserved in at least part of the ALS population and, therefore, present highly usable neuroelectrical signal features that are relevant for BCI control in this population. Indeed, these features have been used to accomplish BCI control in several non-invasive (Wolpaw et al., 1997; Kübler et al., 2005; Bai et al., 2010) and implanted (Vansteensel et al., 2016; Milekovic et al., 2018) BCI studies in people with ALS.

### Brain Stem Stroke

UNP4 showed a clear oscillation between 6 and 10 Hz, but oscillations between 10 and 30 Hz were virtually absent. In addition, none of the LFB frequency ranges showed consistent modulation by attempted hand movement in this LIS participant. One possible explanation for the small or absent modulation in LFB power may be an impaired ability to focus on the task, as a result of the brain stem lesion. Indeed, it has been reported that pontine lesions may lead to a deficit in mental imagery of hand rotation (Conson et al., 2008). However, since the analysis of the LFB oscillatory components was conducted only using runs/electrode pairs that showed a significant HFB response, poor or variable (mental) task performance is unlikely to have caused the lack of clear LFB responses.

It should be considered that atypical baseline LFB oscillations and an inability to generate motor-related changes in LFB power may be generalized features of individuals who suffered from a brain stem stroke. Earlier studies of the effects of brain stem stroke on the baseline neural signal indicate a fairly normal EEG with a clear alpha peak and some slowing in the low frequencies (Chase et al., 1968; Hawkes and Bryan-Smyth, 1974; Markand, 1976; Patterson and Grabois, 1986; Babiloni et al., 2010; Kotchoubey and Lotze, 2013). Indeed, in our study, UNP4 presented a clear 6–10 Hz peak during rest, which is in agreement with these earlier findings. However, our finding that the spectrum of UNP4 does not contain a clear oscillation in the 12–22 Hz range, as opposed to what was typically observed in the epilepsy participants, does not agree with a previous report that showed no difference in beta power between people with brain stem stroke and healthy controls (Babiloni et al., 2010). Unfortunately, existing evidence on the consistency of LFB modulation in people with brain stem stroke is scarce. In one EEG study, a participant who was paralyzed because of a brain stem stroke (but could produce speech and had some control over the upper limbs) used the beta rebound for BCI control (Höhne et al., 2014). In addition, in two brain stem stroke participants of the BrainGate study, reliable offline decoding was accomplished using LFB and HFB power changes induced by attempted arm movements (Perge et al., 2014). Interestingly, both these studies reported that frequent calibration was necessary to maintain reliable decoding, as there was substantial variability in the anatomical location of the beta rebound (Höhne et al., 2014) or in the neural signals (Perge et al., 2014). Taken together, it seems that the characteristics of the LFB features of UNP4 are not entirely representative of the brain stem stroke population.

**FIGURE 9:**
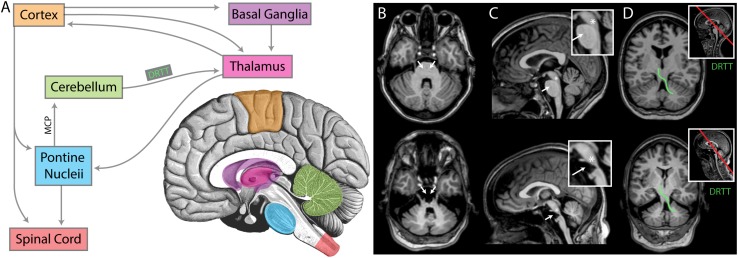
An anatomic interpretation for the lack of beta oscillation in UNP4. **(A)** A simplified diagram of the relevant structures of the motor circuit. MCP denotes the middle cerebral peduncle. DRTT denotes dentatorubrothalamic tract. For panels **(B–D)**, the upper brain images are for participant UNP1 (ALS) and the lower are for participant UNP4 (pontine stroke). **(B)** Axial sections at the level of the MCP. White arrows indicate the location of pontine nuclei, receiving input from cortex and thalamus, and projecting (via the MCP) to the cerebellum. Note the absence of pontine structures in participant UNP4. **(C)** Midsagittal sections, with insets showing magnification of the brainstem. White arrows again indicate the location of pontine nuclei. The asterisks denote the approximate location of the pedunculopontine nucleus (PPN), a structure exhibiting prominent beta oscillations that is involved in movements and locomotion (amongst other modalities) with widespread projections. Note that the PPN appears structurally intact for both participants. **(D)** Sections through the DRTT (shown for one side in green), beginning in the dentate nucleus of the cerebellum, passing through the superior cerebellar peduncle and then the red nucleus, terminating in the ventrolateral nucleus of the thalamus. Section plane shown on midsagittal image with red line in inset. Note that the DRTT appears structurally intact for both participants.

It may be hypothesized that the specific anatomic location of the brain stem damage is related to the presence or absence of cortical oscillation in the beta range. Indeed, beta oscillation has typically been attributed to the corticobasal ganglial-thalamic feedback loop (DeLong and Wichmann, 2007; McCarthy et al., 2011; Miller et al., 2012; Basha et al., 2014). Lack of this feature in the setting of brainstem stroke suggests that the wider motor circuit plays a role (Figure 9). We examined the specific anatomic lesion caused by the brain stem stroke of UNP4. Because both UNP1 and UNP4 exhibit a loss of functional connection with the spinal cord, it is not the likely reason for the virtual absence of oscillations in the beta range in only UNP4. The pedunculopontine nucleus (PPN) has reciprocal connections with cortical, basal ganglial, thalamic, and cerebellar structures, and it exhibits robust beta range oscillations (Shimamoto et al., 2010), making it a reasonable candidate structure. However, the PPN appears to be anatomically preserved in UNP4 (Figure 9). Ascending projections from the cerebellum to the thalamus (dentatothalamic/dentatorubrothalamic tract; Mollink et al., 2016) appear to be intact as well. However, descending cortical and thalamic projections to rostral and caudal pontine nuclei (which, in turn, project to the cerebellum via the middle cerebral peduncle; Evarts and Thach, 1969; Salmi et al., 2009; Kamali et al., 2010; Grimaldi and Manto, 2012) are completely obliterated in UNP4. We therefore consider it most likely that the absence of oscillations in the beta range in UNP4 are the result of the specific location of damage in the motor network.

### Limitations

The present work has several limitations. First is the small number of people with LIS included in the study. It will be necessary to assess whether our findings on the LFB and HFB ECoG features are specific to the participants included here, or whether they present more generalized phenomena intrinsic to the different underlying etiologies of LIS. Yet, given the fact that our findings are corroborated by a substantial amount of data for each of the LIS participants, we believe that the results of this study are valuable for determining the most promising signal features for BCI control in people with LIS.

Second, intrinsic aspects of the reported ECoG data are the sparse sampling of the brain, due to the relatively large inter-electrode distance (1 cm) and the subject-specific electrode locations. As a result, there are inevitable differences in the exact neural populations recorded and used for the analyses across participants. Importantly, the location of the electrodes of UNP1 and UNP4 was driven by the fMRI activation pattern generated by attempted hand movement and the sampled neural populations should therefore be functionally comparable. The most frequently studied electrode pair of both UNP1 and UNP4 included an electrode over the precentral gyrus and one more posterior, over the central sulcus/postcentral gyrus. For the epilepsy participants, we attempted to sample the same functional region as for the LIS participants by using anatomical (the superior part of the sensorimotor cortex), directional (rows perpendicular as possible to the central sulcus) and signal (significant changes in HFB power induced by hand movement) constraints to select the electrode strips for analysis (see Figure 1). In addition, we studied a relatively large population of epilepsy participants. Despite these efforts, it cannot be entirely excluded that differences in the specific electrode locations over the sensorimotor hand area are associated with some level of variability in the LFB and HFB responses.

Third, it should be noted that the analyses conducted here are based on bipolar referenced signals. Since bipolar referencing is the method the implanted device of the LIS participants uses to measure signals from the subdural electrodes, the data of the epilepsy participants was analyzed using similar referencing, to make sure that the data of able-bodied and LIS participants could be accurately compared. It may be speculated that bipolar signals have fundamentally different characteristics than the unipolar signals typically reported in EEG and ECoG literature. In theory, the lack of a typical mu-oscillation in our bipolar ECoG data may even be explained by a complete in-phase synchronization of the area measured by the bipolar pair. However, since the LFB phenomena we observed in the bipolar referenced signals of the epilepsy participants largely corresponded with features observed for single ECoG electrodes in an earlier study (Groppe et al., 2013), we believe that the effect of the bipolar signal processing on our results is of limited significance.

Finally, as the low frequency band was the main focus of the current study, we did not investigate whether or not different aspects of the high-frequency band showed different responses. It will be interesting to investigate this topic in future work.

## Conclusion

Attempted hand movement by two people with LIS generates consistent HFB power changes in the sensorimotor cortex, while baseline oscillations in the low frequencies, and modulation thereof by attempted hand movement, may be substantially affected by the underlying etiology of the motor impairment of people with LIS. These results bear relevance for the development of BCIs for this population, but should be confirmed in larger numbers of individuals.

## Data Availability Statement

The datasets for this study will not be made publicly available because some participants did not consent for public sharing of their data. Part of the data is available upon request.

## Ethics Statement

This study was carried out in accordance with the Declaration of World Medical Association (2013). Epilepsy participants gave written informed consent to participate in the study. LIS participants gave informed consent via a dedicated procedure (see Vansteensel et al., 2016 for details). The protocol was approved by the Medical Research and Ethics Committee Utrecht.

## Author Contributions

ZF, MV, EA, and NR designed the study. TD led the team who designed the implanted device and provided technical support of the BCI system. SL and EP acquired the data. ZF, SL, and MB analyzed the data. ZF and MV drafted the manuscript. All authors interpreted the data and revised the manuscript.

## Conflict of Interest

TD was an employee of Medtronic Inc., which co-sponsored the STW-grant. He is a shareholder of Medtronic and holds patents related to patient directed therapy control (8,380,314), a chopper-stabilized instrumentation amplifier (8,354,881), and a therapy control based on a patient movement state (8,121,694), all licensed to Medtronic. The remaining authors declare that the research was conducted in the absence of any commercial or financial relationships that could be construed as a potential conflict of interest.
